# Supplementary material for: Development of a Comprehensive Quality Evaluation System for Foxtail Millet from Different Ecological Regions
Source: Foods. 2023 Jun 29;12(13):2545. doi: 10.3390/foods12132545 (PMC10340742; doi:10.3390/foods12132545)
Supplement: Supplementary file 1 [file foods-12-02545-s001.zip › Supplementary Table.pdf]

Supplementary Table S1

	Altitude (m)	Longitude	Latitude	pH soil	Available nitrogen (mg/kg)	Available phosphorus (mg/kg)	Available potassium (mg/kg)	Organic matter (g/kg)	Total nitrogen (g/kg)	Total phosphorus (g/kg)	Total potassium (g/kg)
Qinxian	1023	112°39′30″	36°44′2″	8.21	44.58	9.95	195.82	6.85	1.000	0.460	24.84
Zezhou	910	113°1′17″	35°34′31″	8.06	53.50	8.61	194.40	24.44	1.436	0.614	20.78
Dingxiang	764	112°54′56″	38°34′57″	8.26	60.63	13.96	119.40	11.60	0.890	0.630	18.30
Xingxian	1022	110°11′24″	38°11′24″	8.59	21.40	10.90	220.59	12.19	0.530	0.590	23.64
Yuci	1179	112°51′25″	37°49′8″	8.19	53.50	8.42	137.80	15.43	0.785	0.575	15.44

Supplementary Table S2

Standard	Degree of decomposition	Definition
1	Grain unchanged	White core in grain
2	Grain expanded	White core in grain, with powdery ring
3	Grain expanded, with incomplete or narrow ring	White core in grain, with flocculent or nebulous ring
4	Grain enlarged, with complete and wide ring	Cotton white core in grain, with nebulous ring
5	Grain cracked, with complete and wide ring	Cotton white core in grain, with clear ring
6	Grain partially dispersed and dissolved, blended with ring	Cloud white core in grain, with no ring
7	Grain completely dispersed	Both core and ring disappeared in grain

Supplementary Table S3

	max	min	mean	SD	CV (%)
1 000-grain weight (g)	2.80	2.60	2.67	0.08	2.94
Diameter of grain (mm)	1.70	1.56	1.63	0.06	3.58
L*	60.78	58.49	59.57	1.09	1.83
a*	12.30	10.38	11.19	0.70	6.27
b*	49.12	44.32	47.01	1.80	3.82
CCI	4.28	3.78	4.00	0.18	4.54
Moisture content (%)	10.99	10.53	10.72	0.18	1.64
Amylose content (%)	13.36	12.28	12.63	0.43	3.38
Crude fat content (%)	3.92	3.27	3.71	0.25	6.78
Crude protein content (%)	13.23	10.76	12.30	0.94	7.65
Total polyphenols content (mg/100g)	78.03	51.35	62.68	11.01	17.57
Total flavones content (mg/100g)	103.21	45.18	65.71	23.25	35.39
Yellow pigmen content (μg/g)	62.97	54.59	58.61	3.28	5.59
Polysaccharide content (mg/g)	141.81	115.50	122.78	11.03	8.98
Alkali digestion value	4.60	1.17	3.40	1.35	39.75
Gel consistency (mm)	107.29	91.81	98.63	5.58	5.66
Water solubility index (%)	11.00	5.67	8.45	2.02	23.96
Water absorption index (%)	229.33	219.83	225.07	3.65	1.62
Umami amino acids content (%)	3.45	2.98	3.24	0.20	6.08
Sweet amino acids content (%)	2.87	2.50	2.72	0.14	4.96
Bitter amino acids content (%)	5.95	5.36	5.72	0.23	4.03
Different value of amino acids (%)	0.44	0.12	0.25	0.13	50.30

Supplementary Table S4

Clustering	The most representative variable	The least representative variable
1	Gel consistency	Polysaccharide content
2	Crude fat content	Alkali digestion value
3	L*	L*
4	Total polyphenols content	Water absorption index
5	Amylose content	Amylose content
6	Crude protein content	Different value of amino acids

Supplementary Table S5

	Qinxian	Zezhou	Dingxiang	Xingxian	Yuci
X1	0.20	-0.69	1.60	-0.18	-0.94
X2	0.86	-1.20	1.20	-0.34	-0.51
X3	-0.21	1.11	-0.99	0.97	-0.88
X4	-0.27	-0.27	1.59	0.10	-1.15
X5	-0.32	0.51	1.18	0.13	-1.50
X6	-0.03	-1.19	1.57	-0.36	0.02
X7	0.30	-0.22	1.55	-0.56	-1.07
X8	-0.56	1.71	-0.05	-0.28	-0.82
X9	0.33	-1.74	0.85	0.25	0.29
X10	0.09	0.58	-1.64	-0.01	0.98
X11	1.39	0.50	-1.03	0.00	-0.86
X12	1.61	0.10	-0.78	-0.88	-0.05
X13	-0.66	0.54	1.33	0.01	-1.23
X14	-0.53	-0.66	1.73	0.01	-0.55
X15	0.68	-1.65	0.13	-0.06	0.89
X16	-0.28	-0.13	1.55	0.08	-1.22
X17	0.60	-0.11	-1.37	1.26	-0.38
X18	1.17	0.58	0.17	-0.48	-1.43
X19	0.18	0.80	-1.32	-0.70	1.04
X20	0.32	0.25	-1.63	-0.02	1.09
X21	0.11	1.03	-1.53	-0.31	0.70
X22	0.43	-0.37	-1.03	-0.55	1.52
