# Supplementary figures and images for: Development of a Comprehensive Quality Evaluation System for Foxtail Millet from Different Ecological Regions
Source: Foods. 2023 Jun 29;12(13):2545. doi: 10.3390/foods12132545 (PMC10340742; doi:10.3390/foods12132545)

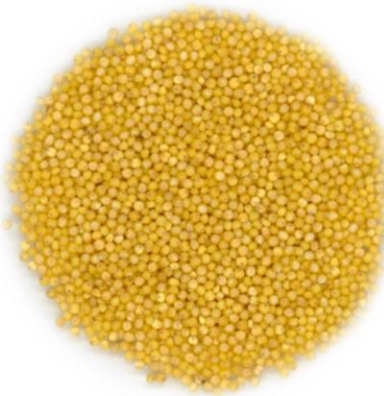

Qinxian

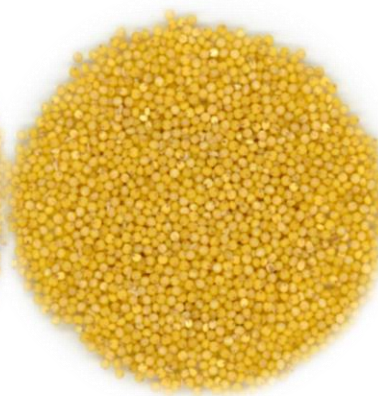

Zezhou

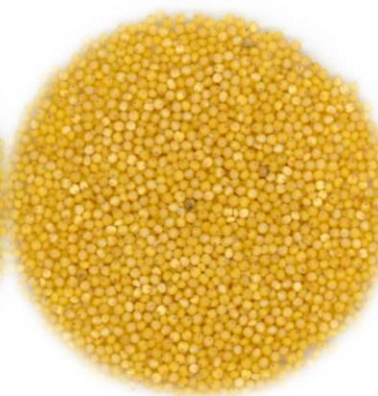

Dingxiang

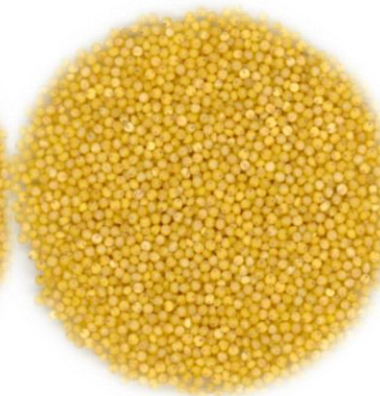

Xingxian

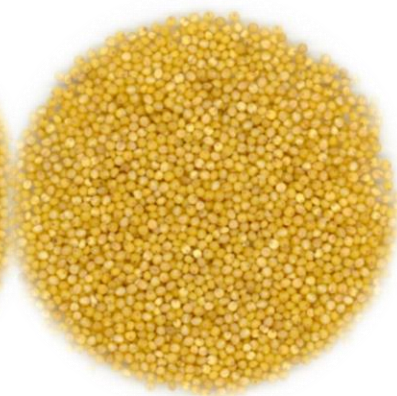

Yuci

Supplement: Supplementary file 1 [file foods-12-02545-s001.zip › Supplementary Figure.pdf]
